# Factors Associated with Perceptions of Maternal Control and Support During Childbirth and Relationship with Childbirth Satisfaction Among Women in Spain

**DOI:** 10.3390/medicina62071281

**Published:** 2026-07-03

**Authors:** Sergio Martínez-Vázquez, Rocío Adriana Peinado-Molina, Leticia Molina-García, Antonio Hernández-Martínez

**Affiliations:** 1 Department of Nursing, University of Jaén, 23071 Jaén, Spain; svazquez@ujaen.es (S.M.-V.); lemolina@ujaen.es (L.M.-G.); 2 Consortium for Biomedical Research in the Epidemiology and Public Health (CIBERESP), 28029 Madrid, Spain; 3 University Hospital of Jaén, 23007 Jaén, Spain; 4 Department of Nursing, Physiotherapy and Occupational Therapy, Ciudad Real, Faculty of Nursing, University of Castilla-La Mancha, 13071 Ciudad Real, Spain; antonio.hmartinez@uclm.es; 5 Research Group on Care and Evidence-Based Practice, Castilla-La Mancha Institute for Health Research (IDISCAM), 13071 Ciudad Real, Spain

**Keywords:** birth plan, internal-external control, patient satisfaction, childbirth experience, peripartum period, perceived control, respectful maternity care

## Abstract

*Background and Objectives*: The World Health Organisation (WHO) promotes respectful, woman-centred care during childbirth, aimed at preserving women’s autonomy, their sense of control and the fulfilment of their expectations. Loss of control during childbirth has been linked to a poorer birth experience and adverse emotional consequences in the postpartum period. To analyse the maternal and perinatal factors associated with perceived control and support during childbirth, and their relationship with childbirth satisfaction in women in Spain. *Materials and Methods*: A cross-sectional study was conducted with postpartum women who had given birth within the previous six months in Spain. Validated instruments were used, including the “Support and Control in Birth” (SCIB) scale and the “Birth Satisfaction Scale-Revised” (BSS-R). Sociodemographic, obstetric and neonatal variables were also collected. Associations were assessed using multiple linear regression models. *Results*: A total of 302 postpartum women participated, with a mean age of 35.2 years (SD = 4.18), of whom 70.2% (*n* = 212) were primiparous. Satisfaction with childbirth (BSS-R) was the strongest positive association with all dimensions of the SCIB: internal control (adjusted mean difference [aMD] = 0.56; 95% CI: 0.48, 0.64), external control (aMD = 0.75; 95% CI: 0.61, 0.89), support (aMD = 0.89; 95% CI: 0.77, 1.01) and overall score (aMD = 2.34; 95% CI: 2.13, 2.56). Greater pain intensity was associated with a lower perception of internal control (aMD = −1.00; 95% CI: −1.31, −0.70). In comparison with adherence to the birth plan, non-adherence showed a negative association with the support dimension (aMD = −7.71; 95% CI: −10.72, −4.71) and with the overall SCIB score (aMD = −10.21; 95% CI: −16.01, −4.42). Women’s active participation in decision-making was positively associated with perceived control and support (aMD = 7.15; 95% CI: 2.21, 12.09). *Conclusion*: A greater perception of support and control during labour is associated with a more positive birth experience. Deviation from the birth plan and greater pain intensity are associated with a poorer perception of control, whilst women’s active participation was associated with higher perceived control. These findings underscore the importance of personalised and respectful care to improve women’s childbirth experience, particularly perceived support, control and satisfaction.

## 1. Introduction

The World Health Organisation (WHO) promotes respectful, woman-centred care during childbirth, aimed at preserving women’s autonomy, their sense of control and the fulfilment of their expectations [1]. A positive birth experience is associated with higher levels of perceived control and satisfaction with the care and support received during pregnancy, childbirth and the postpartum period [2]. Conversely, a negative experience has been linked to fear of childbirth, a higher likelihood of instrumental delivery or caesarean section, and poorer subsequent quality of life [3].

Childbirth experiences perceived as traumatic are not uncommon. In Spain, their prevalence has been estimated at between 11% and 20% [4,5], whilst globally it can reach 30% [6]. In the specialized literature, a perceived loss of control and poor relational support during childbirth are consistently identified as the primary drivers of these negative subjective experiences [5,6]. While severe cases can ultimately escalate into postpartum psychological sequelae such as PTSD or depression [6,7], research increasingly focuses on the immediate childbirth experience itself, showing that maternal satisfaction is directly shaped by how a woman navigates the balance between clinical interventions and personal agency [7].

To optimize this experience, birth plans—understood as documented birth preferences rather than guaranteed clinical outcomes—were developed to improve informed decision-making by women and communication between women and healthcare professionals. In existing research, the fulfillment or respectful handling of these documented preferences is heavily correlated with enhanced patient satisfaction and a protected sense of maternal control, whereas ignoring them often triggers a perceived loss of autonomy [8].

The childbirth experience is determined by multiple factors, including perceived support, internal control and obstetric outcomes [5,9]. Although the perception of maternal control is a subjective construct and difficult to measure objectively, previous studies have shown that a greater sense of control is associated with greater satisfaction and better obstetric outcomes [10]. This perception includes both internal control, related to women’s ability to manage their emotions, behaviour and pain during childbirth, and external control, related to the information received, decision-making, the birth environment and the care provided by healthcare professionals [11] (In quantitative studies, these specific dimensions do not act in isolation; rather, high levels of external care and professional support have been shown to directly reinforce a woman’s internal coping capacity and overall satisfaction during labor [5,12,13].

The perception of control during childbirth is underpinned, in part, by internal locus of control, understood as the belief that events depend on one’s own capacity for action [4,14]. This component constitutes an important foundation of maternal satisfaction and is closely related to self-esteem. Its loss has been associated with lower self-esteem and with negative emotions and self-perceptions [15,16,17,18,19]. However, the perception of support and control during childbirth is a complex, multifactorial phenomenon, modulated by sociodemographic and clinical variables. The literature indicates that these background factors heavily influence a woman’s baseline expectations and choices; for instance, higher socioeconomic status, the targeted use of epidural analgesia, an un-medicalized onset of labour, or a negative history of mental health care can significantly alter how support is evaluated and how control is maintained throughout the obstetric trajectory [5,9].

However, although several factors have been related to childbirth satisfaction and perceived control, evidence on the maternal and perinatal factors independently associated with the specific dimensions of support and control during childbirth remains limited, particularly in the Spanish context. In this regard, the recent validation of the SCIB scale in Spanish postpartum women allows the assessment of internal control, external control and support using a specific instrument adapted to this population. Based on the existing literature, it may be expected that childbirth satisfaction, respected birth preferences and active participation are positively related to perceived support and control, whereas clinical or psychosocial vulnerability factors may be associated with lower scores.

The present study extends the use of the SCIB scale by examining the maternal and perinatal factors associated with perceived support and control during childbirth and their relationship with childbirth satisfaction. In this context, the aim of the present study was to identify and quantify the maternal and perinatal factors independently associated with the dimensions of support and control during childbirth, as measured by the “Support and Control in Birth” (SCIB) scale [20], and their relationship with childbirth satisfaction in a sample of postpartum women in Spain. Understanding these factors may help to guide personalised and evidence-based care strategies aimed at improving women’s childbirth experience.

## 2. Methodology

*Study design and selection criteria*

A cross-sectional study was conducted in Spain with women in the postpartum period. Women aged 18 years or older who had given birth in the 6 months prior to data collection and who understood Spanish were included. Minors and women with a language barrier preventing adequate understanding of the questionnaire or the informed consent form were excluded. Adequate understanding was verified by the collaborating healthcare professional during the information and consent process, based on the woman’s ability to understand the information provided in Spanish and to complete the questionnaire independently. All questionnaires were completed during the postpartum period, within six months after delivery.

*Data collection and procedures*

Participants were recruited through collaborating healthcare professionals in various clinical settings, including postnatal wards, delivery rooms, and midwifery clinics in health centers. This strategy enabled the identification of potential participants both during hospital admission and during subsequent maternal and child follow-up.

Once eligibility criteria had been verified, women received verbal and written information about the study. Those who agreed to participate signed the informed consent form and received instructions on how to complete the questionnaire. To address any procedural queries related to participation in the study, participants had the support of the healthcare professional and access to a communication channel set up specifically for this purpose. Data collection was split into clinical extraction and participant self-report. Obstetric and perinatal variables were extracted from the institutional electronic medical records by collaborating midwives and obstetricians using a standardized data extraction sheet. Concurrently, the psychological and satisfaction instruments (SCIB and BSS-R) were distributed by the collaborating clinicians but completed independently by the participants via a self-administration format to minimize response bias. Additional complementary sociodemographic and childbirth-related information not covered by the validated instruments was gathered using a previously piloted ad hoc questionnaire, which was also completed independently by the participants.

*Variables and Instrumentation*

The data were collected by reviewing medical records and using a previously piloted ad hoc questionnaire designed to collect complementary sociodemographic and childbirth-related information not covered by the validated instruments. Sociodemographic, obstetric and perinatal variables were collected. Sociodemographic variables included, amongst others, age, educational level, employment status and other maternal contextual characteristics. Obstetric variables included obstetric history, gestational risk, onset of labour, mode of delivery, use of epidural analgesia, pain intensity, the woman’s active participation during labour, adherence to the birth plan, and history of mental health care. Active participation during childbirth was recorded according to the woman’s self-reported involvement in the birth process and decision-making, and was classified as: yes, no by choice, or no because it was not possible. Birth preferences were classified as no documented birth preferences, preferences respected, or preferences not respected, according to the woman’s report. Health problems during pregnancy were recorded as present or absent, based on self-report and information from the clinical record.

The primary outcome variable was the “Support and Control in Birth” (SCIB) scale, a validated 33-item instrument that assesses maternal perception across three dimensions: internal control, external control and support received during labour. The internal control dimension includes aspects related to emotions, thoughts, behaviour, pain and physical functioning; the external control dimension covers pain relief, information, the birth environment, decision-making and outcomes; and the support dimension assesses guidance, the attitude of healthcare staff, empathy, encouragement and assistance with pain relief. The scale was administered once during the postpartum period. In its validation within a similar population, the SCIB demonstrated high internal consistency (Cronbach’s alpha = 0.951) [20].

The “Birth Satisfaction Scale-Revised” (BSS-R) was also included, a 10-item instrument that assesses satisfaction with the childbirth experience across three subscales: quality of care received, personal attributes of the woman, and stress experienced during childbirth. The validated Spanish version showed adequate internal consistency (Cronbach’s alpha = 0.770) [21].

*Statistical analysis*

*Sample size estimation*

The sample size was estimated for multiple linear regression models, considering the continuous nature of the primary outcomes, namely the total SCIB score and its three dimensions. Assuming a maximum of 15 predictors in the multivariable models, an alpha level of 0.05, a statistical power of 80%, and a small-to-moderate effect size for the overall regression model, a minimum sample size of approximately 200 participants would be required to detect an effect size of f^2^ = 0.10. The final sample included 302 women, exceeding this requirement and allowing sufficient precision for the estimation of adjusted associations. In addition, with 302 participants, 15 predictors and alpha = 0.05, the study had 80% power to detect an effect size of approximately f^2^ = 0.065, which is between a small and moderate effect according to conventional criteria.

*Descriptive and Inferential Modeling*

Qualitative variables were described using absolute and relative frequencies, and quantitative variables using mean and standard deviation (SD). Subsequently, a bivariate analysis was performed between the maternal variables collected and the three dimensions of the SCIB scale, as well as its total score. The results were expressed as mean differences (MD) with their 95% confidence intervals (95% CI).

To identify factors independently associated with the perception of support and control during childbirth, multiple linear regression models were constructed using the scores of the SCIB sub-dimensions and their overall score as dependent variables. The results were expressed as adjusted mean differences (aMD) with their respective 95% CI. The selection of covariates for the multiple linear regression models was based on clinical relevance and on statistically significant associations observed in the bivariate analysis. Separate models were constructed for each SCIB dimension and for the overall SCIB score. Given the cross-sectional design of the study, the results were interpreted as adjusted associations. Missing data were handled using complete-case analysis for each regression model.

*Model Diagnostics and Sensitivity Analyses*

Global model fit was assessed using R, R^2^, adjusted R^2^, the standard error of the estimate and the overall F test. The assumptions of multiple linear regression were evaluated before interpreting the models. Independence of residuals was assessed using the Durbin-Watson statistic. Multicollinearity was examined using tolerance values and variance inflation factors. Residual diagnostics included standardised residuals, studentised residuals, Mahalanobis distance, centred leverage values and Cook’s distance to identify potential outliers or influential observations. Cook’s distance values below 1 were considered to indicate the absence of highly influential observations. Two sensitivity analyses were performed to assess the robustness of the main findings. First, the multiple linear regression models were repeated after restricting the sample to women who had a vaginal birth, in order to explore whether the main associations were maintained in a more clinically homogeneous subgroup. Second, the models were repeated after excluding the BSS-R score, given the conceptual proximity between childbirth satisfaction and perceived support and control during childbirth. These sensitivity analyses were interpreted cautiously because of the reduced sample size in the vaginal birth subgroup and the exploratory nature of the additional models. Statistical analysis was performed using SPSS version 29.0.

*Ethical considerations*

The study was conducted in accordance with the principles of the Declaration of Helsinki and current regulations governing research involving human subjects. All participants were informed about the study objectives, the voluntary nature of their participation and data confidentiality, and signed an informed consent form prior to inclusion. Data were treated confidentially and anonymised prior to analysis. The study was approved by the Research Ethics Committee of the Province of Jaén (SICEIA-2024-000229).

## 3. Results

The results are presented according to the analytical sequence followed in the study: descriptive analysis, bivariate analysis and multiple linear regression models.

A total of 302 postpartum women participated, with a mean age of 35.2 years (SD = 4.18). The majority were primiparous (70.2%; n = 212), whilst 29.8% (n = 90) were multiparous. Regarding the birth plan, 35.4% (n = 107) indicated that it was followed, and 12.6% (n = 38) reported that, despite having submitted one, it was not followed. The mean pain intensity during labour was 7.54 points (SD = 2.44) on a scale of 0 to 10, and the mean number of visits to A&E due to pregnancy complications was 1.50 (SD = 1.89). 34.4% (n = 104) were highly satisfied with their antenatal care, whilst 44.0% (n = 133) were satisfied with the care received during the postnatal period. 59.9% (n = 181) had a vaginal delivery, 82.5% (n = 249) used epidural analgesia, and 22.2% (n = 67) reported that they were unable to participate actively during labour. The remaining sociodemographic and clinical characteristics of the sample are presented in Table 1. Descriptive statistics for the main study instruments are presented in Table 2. The mean BSS-R total score was 26.68 (SD = 9.00), while the mean total SCIB score was 113.86 (SD = 27.91). Mean scores for the SCIB dimensions were 34.18 (SD = 8.68) for internal control, 35.31 (SD = 11.83) for external control and 44.37 (SD = 12.27) for support.

In the bivariate analysis, statistically significant associations were observed between various maternal and obstetric factors and the dimensions of support and control during labour as measured by the SCIB scale. Monthly income was associated with the internal control dimension (*p* = 0.002) and with the total SCIB score (*p* = 0.040). High-risk pregnancy was associated with lower scores on the dimensions of internal control (*p* = 0.002), external control (*p* < 0.001), support (*p* = 0.006) and on the overall SCIB score (*p* < 0.001). Furthermore, the onset of labour, the birth plan, the mode of delivery, postpartum complications, the need for special care for the newborn, the woman’s active participation during labour, skin-to-skin contact and early initiation of breastfeeding showed statistically significant associations with the three dimensions of the SCIB and with its total score. The full details of the bivariate analysis are presented in Table 3.

In the multiple linear regression models, satisfaction with childbirth as measured by the BSS-R was the strongest positive predictor of all dimensions of the SCIB and its overall score. For every additional point on the BSS-R, statistically significant increases were observed in internal control (aMD = 0.56; 95% CI: 0.48, 0.64), external control (aMD = 0.75; 95% CI: 0.61, 0.89), support (aMD = 0.89; 95% CI: 0.77, 1.01) and the total SCIB score (aMD = 2.34; 95% CI: 2.13, 2.56).

With regard to pain intensity during labour, each additional point was associated with a lower score on the internal control dimension (aMD = −1.00; 95% CI: −1.31, −0.70). Furthermore, high-risk pregnancy, compared with low-risk pregnancy, was associated with a lower perception of support (aMD = −0.75; 95% CI: −1.39, −0.10) and a lower overall score on the SCIB. Similarly, compared with women whose birth plan was respected, those whose birth plan was not respected had significantly lower scores on the support dimension (aMD = −7.71; 95% CI: −10.72, −4.71) and in the overall SCIB score (aMD = −10.21; 95% CI: −16.01, −4.42).

Furthermore, active participation during childbirth was also found to be significantly associated with a greater sense of control and support. In particular, compared with women who were unable to participate actively, those who did participate scored higher on the external control dimension (aMD = 7.15; 95% CI: 2.21, 12.09). In contrast, previous mental health care was also associated with lower external control scores (aMD = −2.40; 95% CI: −4.14, −0.67). Other variables associated with any of the SCIB dimensions or with the overall score included a history of mental health care, health problems during pregnancy, religiosity, use of epidural analgesia and type of delivery. The size of these associations should also be taken into account, particularly for satisfaction with childbirth and non-adherence to the birth plan, as the differences observed in the overall SCIB score may be relevant for women’s perception of support and control during childbirth. All multiple linear regression models were statistically significant. The adjusted R^2^ values were 0.461 for internal control, 0.622 for external control, 0.594 for support and 0.701 for the overall SCIB score. Durbin-Watson values ranged from 1.88 to 2.02, suggesting no relevant autocorrelation of residuals. Multicollinearity was generally low in the total SCIB, internal control and support models. In the external control model, two dummy variables related to active participation showed VIF values above 5, probably reflecting the imbalance between response categories; therefore, these specific coefficients were interpreted with caution. Although some standardised residuals exceeded ±3, no highly influential observations were identified, as all Cook’s distance values were below 1. The remaining results are presented in Table 4. The sensitivity analyses are presented in Supplementary Tables S1 and S2. In the analysis restricted to women with vaginal birth, all models remained statistically significant. The adjusted R^2^ values were 0.561 for the overall SCIB score, 0.436 for internal control, 0.438 for external control and 0.402 for support. The BSS-R score remained positively associated with all SCIB dimensions and with the overall SCIB score. Pain intensity during labour remained negatively associated with internal control, and unmet documented birth preferences remained negatively associated with the support dimension. When the models were repeated after excluding the BSS-R score, all models also remained statistically significant, although the adjusted R^2^ values decreased, as expected. The adjusted R^2^ values were 0.227 for the overall SCIB score, 0.145 for internal control, 0.462 for external control and 0.274 for support. In these models, unmet documented birth preferences were strongly associated with lower support and lower overall SCIB scores. Pain intensity remained negatively associated with internal control, previous mental health care was associated with lower external control, and high-risk pregnancy was associated with a lower overall SCIB score. Overall, both sensitivity analyses supported the direction of the main findings, while showing that some estimates varied in magnitude and statistical significance depending on the model specification and subgroup analysed.

## 4. Discussion

The findings of this study show that a greater perception of support and control during childbirth is associated with a more positive overall experience. In particular, satisfaction with childbirth showed the strongest positive association with the support and control dimensions assessed, whilst severe pain, high-risk pregnancy and, in particular, non-adherence to the birth plan were associated with a poorer perception of support and control. Furthermore, the woman’s active participation during childbirth was associated with a greater perception of control. Taken together, these results reinforce the multifactorial nature of the childbirth experience and highlight the influence of clinical, contextual and psychosocial factors.

Furthermore, the combined use of clinical records and previously piloted questionnaires may have helped to reduce recall bias, at least in relation to clinical and obstetric variables. Among the study’s main strengths is its innovative nature, as it comprehensively analyses the perception of support and control during childbirth using a tool recently validated in the Spanish context [20], together with obstetric and neonatal variables and mental health history. This comprehensive approach allows for a broader understanding of the childbirth experience, moving beyond models focused exclusively on obstetric indicators.

Our results are consistent with previous research indicating that a greater perception of support and control is associated with a more positive childbirth experience and may be associated with fewer negative experiences and potential emotional consequences [22]. These findings are particularly relevant, as they position perceived support and maternal control not only as subjective dimensions of the experience, but also as components that may be relevant to postpartum psychological well-being.

Pain during childbirth showed a negative association with the perception of maternal control, suggesting that it should not only be considered a physical symptom, but also a central element in the emotional experience of childbirth. Ford et al. [11] noted that the ability to cope with pain influences perceived control during childbirth, whilst other studies have described how a greater sense of control is associated with lower levels of reported pain and more positive emotions during the process [23,24]. In this regard, our results reinforce the need to monitor and address both pain and the perception of control in a comprehensive manner, given the close interaction between the two. This association may reflect that woman with higher pain levels perceived greater external support, information or pain-relief measures, whereas internal control was more closely related to their ability to cope with pain during labour. This distinction may help to explain why pain can affect different dimensions of perceived control in different ways.

One of the most significant findings of the study was the association between non-adherence to the birth plan and the perception of support. Women whose birth plan was not followed scored significantly lower on this dimension, suggesting that a mismatch between maternal expectations and the care received can significantly impair the birth experience. This result is consistent with previous studies that have shown that the existence of a birth plan and, in particular, adherence to it, are associated with a greater sense of support and a better overall experience [25]. Crucially, from a clinical perspective, non-adherence to a birth plan is rarely an isolated choice by care providers; rather, it is often a proxy for sudden obstetric complications, emergency interventions, or escalating medical issues that inherently necessitate deviations from the original plan. In our multivariable analysis, even when accounting for high-risk status and mode of delivery, this non-adherence retained an independent negative association. This suggests that while medical complications drive the clinical need to deviate from a plan, the subsequent drop in perceived support may stem from how those sudden changes are communicated to the woman during acute stress. In this context, birth planning should not be understood as a guarantee of specific procedures or outcomes, but as a tool to support communication, informed consent and shared decision-making according to maternal and fetal safety.

Similarly, the woman’s active participation during childbirth was associated with a greater sense of control. This finding is consistent with previous literature, which has described maternal involvement in decision-making as a key factor associated with autonomy, empowerment and a more positive birth experience. Active participation may be associated with a more positive subjective experience of childbirth [26,27,28] and may also constitute a distinguishing feature of the quality of care.

On the other hand, women with high-risk pregnancies perceived less support during the process, suggesting that greater clinical complexity is not always accompanied by a more favourable care experience. Although these women tend to receive more intensive care from a biomedical perspective, this does not necessarily translate into greater emotional support or a perception of more respectful care. In this regard, the definition of a positive birth experience proposed by Leinweber et al. [29], based on feeling supported, in control, safe and respected, is particularly relevant in reminding us that the quality of care must be maintained even, or especially, in contexts of higher obstetric risk.

A history of mental health problems was also found to be associated with perceptions of support and satisfaction during childbirth. This finding is particularly relevant, given that some of the existing literature on the childbirth experience excludes women with pre-existing mental health conditions, thereby limiting our understanding of a potentially more vulnerable group [30,31]. The perinatal period is a time of particular psychological vulnerability, during which new disorders may emerge or pre-existing conditions may worsen [32]. Therefore, ensuring a positive birth experience that is sensitive to the emotional needs of these women should be considered a clinical priority, especially in the immediate postpartum period, when the risk of psychological distress may be higher [33].

Finally, our results suggest that obstetric variables such as the onset of labour, the use of epidural analgesia and the mode of delivery also influence perceptions of support, control and satisfaction. Although some authors have described a limited impact of obstetric interventions considered in isolation on the birth experience [34], their own results show poorer experiences among women who underwent caesarean section or had unplanned labour onset, compared with those who had spontaneous vaginal births. Consequently, rather than focusing exclusively on the intervention itself, it seems necessary to explore in greater depth how these circumstances interact with perceptions of autonomy, support and control. Thus, the role of epidural analgesia, mode of delivery and onset of labour should be interpreted in relation to how women felt informed, supported and involved in decision-making. These findings suggest that future studies should evaluate whether care strategies focused on communication, shared decision-making and professional support are associated with better childbirth experiences and subsequent psychological outcomes.

## 5. Limitations and Clinical Implications

This study has some limitations that should be considered when interpreting the results. Firstly, its cross-sectional design does not allow causal relationships to be established. In addition, although the BSS-R and SCIB assess different constructs, they evaluate related dimensions of the childbirth experience. This should be considered when interpreting the strong association observed between childbirth satisfaction and perceived support and control. Moreover, the direction of this association cannot be established, as women who perceived greater support and control during childbirth may also have reported higher childbirth satisfaction. Secondly, although recall bias was minimised through the use of medical records and data collection relatively close to the time of delivery, some self-reported variables may be subject to retrospective assessment. In addition, the recruitment procedure should be considered when interpreting the representativeness of the sample, as some degree of selection bias cannot be ruled out. Furthermore, the sample included only women who understood Spanish, which may limit the generalisability of the findings to more diverse populations. Although several maternal and perinatal variables were included in the models, residual confounding cannot be excluded. Potential multicollinearity was also assessed in the statistical analysis.

Thirdly, while the study restricted participation to the first six months postpartum, the exact timing and setting of questionnaire completion were not perfectly uniform. Potential participants were identified across a spectrum of clinical environments—ranging from acute hospital wards to outpatient midwifery clinics. Consequently, variations in time elapsed since delivery and the immediate physical environment could intersect with transient maternal states, such as acute post-birth physical fatigue, baby-blues, or postpartum stress, potentially impacting the retrospective appraisal of childbirth support and control. Future research should utilize tightly standardized longitudinal intervals to minimize this environmental and temporal variance. Despite these limitations, the results have relevant clinical implications. Identifying factors associated with perceived support and control, such as adherence to the birth plan, the woman’s active participation and appropriate pain management, may help guide strategies aimed at promoting more respectful and woman-centred childbirth care. Similarly, the findings support the need for personalised care, particularly for women with high-risk pregnancies or a history of mental health problems, as these factors may be relevant to women’s childbirth experience and postpartum care.

## 6. Conclusions

Perceived support and control during childbirth were central and interrelated dimensions of childbirth experience. In this sample, higher childbirth satisfaction, active participation in decision-making and respected documented birth preferences were associated with higher perceived support and control, whereas greater pain intensity, high-risk pregnancy and unmet documented birth preferences were associated with lower scores in some SCIB dimensions. These findings highlight the relevance of respectful, personalised and woman-centred care focused on communication, shared decision-making and emotional support during childbirth. Longitudinal studies are needed to confirm these associations and to evaluate their potential relationship with subsequent maternal psychological outcomes.

## Figures and Tables

**Table 1:** Sociodemographic, obstetric and perinatal characteristics of the study sample.

Variable	Total	Variable	Total
	**N (%)**		**N (%)**
**Maternal age**		**Number of pregnancy visits**	
Mean (SD)	35.2 (4.18)	<7	95 (31.5)
**A&E Visits (pregnancy-related)**		7–10	138 (45.7)
Mean (SD)	1.50 (1.89)	>10	69 (22.8)
**Pain level (during birth)**		**Antenatal classes**	
Mean (SD)	7.54 (2.44)	No	70 (23.2)
**Belongs to a religion**		Yes	232 (76.8)
No	148 (49.0)	**Health issue while baby in utero**	
Yes, but not practicing	104 (34.4)	No	277 (91.7)
Yes, and practicing	50 (16.6)	Yes	25 (8.3)
**Current illness**		**Satisfaction degree with pregnancy follow-up**	
No	241 (79.8)	Not at all	5 (1.7)
Yes	61 (20.2)	Low satisfaction	31 (10.3)
**Current medical treatment**		Satisfied	85 (28.1)
No	242 (80.1)	Quite satisfied	77 (25.5)
Yes	60 (19.9)	Highly satisfied	104 (34.4)
**Stressful event during last year**		**Worked during last pregnancy**	
No	189 (62.6)	No	50 (16.6)
Yes	113 (37.4)	Yes	252 (83.4)
**Any previous mental health treatment**		**Health issues during last pregnancy**	
No	125 (41.4)	No	183 (60.6)
Yes	177 (58.6)	Yes	119 (39.4)
**Admission to a mental health unit**		**Birth plan**	
No	298 (98.7)	No	157 (52.0)
Yes	4 (1.3)	Yes, respected	107 (35.4)
**Stillbirth (previous)**		Yes, but not respected	38 (12.6)
No	296 (98.00)	**Onset of last labour**	
Yes	6 (2.0)	Spontaneous	139 (46.0)
**Wanted pregnancy**		Stimulated	141 (46.7)
No	29 (9.6)	Elective C/S	12 (4.0)
Yes	273 (90.4)	Emergency C/S	10 (3.3)
**IVF Assistance (last pregnancy)**		**Type of birth**	
No	268 (88.7)	Vaginal	181 (59.9)
Yes	34 (11.3)	Instrumental	55 (18.2)
**Pregnancy Risk**		Elective C/S	15 (5.0)
Low risk	227 (75.2)	Emergency C/S	51 (16.9)
High risk	75 (24.8)	**Epidural**	
**Monthly income (€)**		No, maternal choice	31 (10.3)
<1000	9 (3.0)	No, medical reason	4 (1.3)
1000–1999	64 (21.2)	No, other reasons	18 (6.0)
2000–2999	90 (29.8)	Yes	249 (82.5)
>3000	139 (46.0)	**Active participation during birth**	
**Skin to skin**		No, by choice	9 (3.0)
No	38 (12.6)	No, not possible	67 (22.2)
Yes	264 (87.4)	Yes	226 (74.8)
**Parity**		**Postpartum complications**	
Primiparous	212 (70.2)	No	249 (82.5)
Multiparous	90 (29.8)	Yes	53 (17.5)
**Any health issue present in last baby born**		**Satisfaction degree with postpartum care**	
No	253 (83.8)	Not at all	7 (2.3)
Yes	49 (16.2)	Low satisfaction	28 (9.3)
**Neonate needed special care**		Satisfied	54 (17.9)
No	263 (87.1)	Quite satisfied	80 (26.5)
Yes	39 (12.9)	Highly satisfied	133 (44.0)
**Early Breastfeeding**			
No	64 (21.2)		
Yes	238 (78.8)		

**Table 2:** Descriptive statistics of childbirth satisfaction and perceived support and control scores.

Instrument/Dimension	n	Mean	SD	Minimum	Maximum
BSS-R total	302	26.68	9.00	2	40
SCIB total	302	113.86	27.91	36	163
Internal control	302	34.18	8.68	10	50
External control	302	35.31	11.83	11	55
Support	302	44.37	12.27	12	60

*BSS-R = Birth Satisfaction Scale-Revised; SCIB = Support and Control in Birth; SD = standard deviation.*

**Table 3:** Comparative bivariate analysis of SCIB scores according to maternal, obstetric and perinatal variables.

SCIB Dimensions				
Variable	Internal Control Mean (SD)	External Control Mean (SD)	Support Mean (SD)	SCIB Total Mean (SD)
**Belongs to a religion**				
No	33.93 (7.90)	34.57 (11.39)	44.84 (11.55)	113.35 (25.59)
Yes, but not practicing	34.76 (9.19)	36.51 (11.97)	45.04 (12.58)	116.31 (28.76)
Yes, and practicing	33.72 (9.82)	35.00 (12.81)	41.58 (13.52)	110.30 (32.51)
***p-value***	0.698	0.434	0.211	0.437
**Monthly income (€)**				
<1000	34.89 (11.77)	41.00 (12.19)	47.78 (15.86)	123.67 (37.69)
1000–1999	30.53 (9.47)	32.69 (12.08)	42.23 (13.02)	105.45 (29.41)
2000–2999	35.72 (8.99)	34.71 (11.21)	44.40 (12.62)	114.83 (27.77)
>3000	34.82 (7.43)	36.53 (11.91)	45.12 (11.42)	116.47 (26.04)
***p-value***	**0.002**	0.072	0.371	**0.040**
**Current illness**				
No	34.28 (8.87)	35.33 (11.83)	44.27 (12.18)	113.88 (27.85)
Yes	33.79 (7.96)	35.23 (11.92)	44.77 (12.73)	113.79 (28.40)
***p-value***	0.691	0.952	0.776	0.981
**Parity**				
Primiparous	34.12 (8.97)	34.40 (11.90)	44.07 (12.45)	112.58 (28.00)
Multiparous	34.32 (8.01)	37.46 (11.43)	45.09 (11.89)	116.87 (27.61)
***p-value***	0.855	**0.040**	0.509	0.224
**Stillbirth (previous)**				
No	34.19 (8.70)	35.34 (11.85)	44.50 (12.19)	114.02 (27.83)
Yes	34.00 (8.63)	34.00 (11.24)	38.17 (15.85)	106.16 (33.69)
***p-value***	0.959	0.784	0.212	0.496
**Wanted pregnancy**				
No	30.48 (10.25)	32.03 (13.18)	37.79 (15.05)	100.31 (36.12)
Yes	34.56 (8.42)	35.66 (11.65)	45.07 (11.76)	115.30 (26.57)
***p-value***	**0.016**	0.117	**0.002**	**0.006**
**IVF Assistance (last pregnancy)**				
No	34.23 (8.67)	35.79 (11.67)	44.51 (12.07)	114.53 (27.65)
Yes	33.82 (8.91)	31.52 (12.57)	43.24 (13.94)	108.59 (29.79)
***p-value***	0.799	0.048	0.568	0.243
**Pregnancy Risk**				
Low risk	35.06 (8.15)	36.79 (11.33)	45.48 (11.48)	117.32 (25.79)
High risk	31.53 (9.70)	30.84 (12.25)	41.03 (13.96)	103.40 (31.48)
***p-value***	**0.002**	**<0.001**	**0.006**	**<0.001**
**Worked during last pregnancy**				
No	31.42 (9.46)	34.32 (12.16)	42.36 (13.45)	108.10 (30.59)
Yes	34.73 (8.43)	35.51 (11.77)	44.77 (12.01)	115.01 (27.27)
***p-value***	**0.014**	0.517	0.205	0.110
**Health issues during last pregnancy**				
No	35.03 (8.35)	36.01 (11.45)	44.81 (11.49)	115.85 (26.49)
Yes	32.88 (9.04)	34.24 (12.36)	43.70 (13.41)	110.82 (29.83)
***p-value***	**0.036**	0.203	0.443	0.126
**Onset of last labour**				
Spontaneous	36.27 (7.96)	39.73 (10.47)	47.85 (10.27)	123.84 (23.67)
Stimulated	32.70 (8.86)	32.83 (11.54)	42.58 (13.03)	108.11 (28.56)
Elective C/S	33.17 (9.34)	24.58 (8.55)	33.00 (10.49)	90.75 (20.89)
Emergency C/S	27.40 (8.19)	21.70 (9.02)	34.90 (11.53)	84.00 (22.11)
***p-value***	**<0.001**	**<0.001**	**<0.001**	**<0.001**
**Antenatal classes**				
No	34.14 (8.62)	35.67 (12.69)	44.57 (11.90)	114.39 (28.44)
Yes	34.19 (8.72)	35.20 (11.58)	44.31 (12.41)	113.71 (27.81)
***p-value***	0.966	0.772	0.876	0.859
**Birth plan**				
No	34.17 (8.75)	35.81 (11.45)	45.08 (11.42)	115.06 (26.74)
Yes, respected	35.64 (8.21)	38.71 (9.74)	48.67 (8.05)	123.02 (20.04)
Yes, but not respected	30.16 (8.60)	23.68 (11.84)	29.32 (14.20)	83.16 (30.94)
***p-value***	**0.004**	**<0.001**	**<0.001**	**<0.001**
**Type of birth**				
Vaginal	35.93 (8.14)	40.19 (9.76)	48.54 (9.92)	124.66 (22.83)
Instrumental	31.33 (9.16)	28.35 (10.84)	38.89 (13.33)	98.56 (27.92)
C/S Elective	34.00 (8.66)	25.87 (8.72)	34.93 (11.05)	94.80 (22.45)
C/S Emergency	31.12 (8.61)	28.27 (11.56)	38.27 (12.87)	97.67 (27.84)
***p-value***	**<0.001**	**<0.001**	**<0.001**	**<0.001**
**Epidural**				
No, maternal choice	38.23 (8.22)	42.87 (9.28)	48.65 (10.44)	129.74 (21.78)
No, medical reason	38.00 (7.79)	44.75 (5.85)	50.75 (7.80)	133.50 (12.01)
No, other reasons	32.17 (7.01)	32.89 (11.25)	45.56 (9.59)	110.61 (21.78)
Yes	33.76 (8.74)	34.39 (11.85)	43.65 (12.61)	111.81 (28.48)
***p-value***	**0.028**	**<0.001**	0.118	**0.003**
**Health issue while baby in utero**				
No	34.40 (8.70)	35.99 (11.68)	44.83 (11.99)	115.22 (27.53)
Yes	31.72 (8.18)	27.80 (11.06)	39.32 (14.41)	98.84 (28.33)
***p-value***	0.139	**0.001**	**0.031**	**0.005**
**Postpartum complications**				
No	34.80 (8.54)	36.36 (11.50)	45.37 (11.85)	116.53 (26.44)
Yes	31.26 (8.84)	30.40 (12.19)	39.68 (13.22)	101.34 (31.34)
***p-value***	**0.007**	**0.001**	**0.002**	**<0.001**
**Neonate needed special care**				
No	34.80 (8.57)	35.92 (11.72)	44.95 (12.05)	115.67 (27.40)
Yes	30.03 (8.38)	31.23 (11.89)	40.46 (13.18)	101.72 (28.66)
***p-value***	**0.001**	**0.021**	**0.033**	**0.003**
**Any health issue present in last baby born**				
No	34.39 (8.69)	35.50 (11.75)	44.73 (12.20)	114.62 (27.82)
Yes	33.10 (8.64)	34.33 (12.29)	42.53 (12.60)	109.96 (28.34)
***p-value***	0.342	0.525	0.252	0.285
**Active participation during birth**				
No, by choice	34.00 (10.09)	33.56 (16.19)	43.78 (13.54)	111.33 (34.42)
No, not possible	28.63 (8.69)	22.37 (8.45)	33.06 (13.22)	84.06 (23.36)
Yes	35.84 (7.94)	39.22 (9.57)	47.75 (9.70)	122.80 (22.25)
***p-value***	**<0.001**	**<0.001**	**<0.001**	**<0.001**
**Skin to skin**				
No	27.26 (8.62)	26.13 (11.77)	35.39 (13.38)	88.79 (29.94)
Yes	35.18 (8.24)	36.63 (11.25)	45.66 (11.57)	117.47 (25.72)
***p-value***	**<0.001**	**<0.001**	**<0.001**	**<0.001**
**Early breastfeeding**				
No	31.77 (9.36)	29.09 (12.06)	39.55 (13.00)	100.41 (29.73)
Yes	34.83 (8.39)	36.98 (11.21)	45.67 (11.76)	117.48 (26.32)
***p-value***	**0.012**	**<0.001**	**<0.001**	**<0.001**
**Stressful event during last year**				
No	35.04 (8.56)	36.67 (11.60)	45.20 (11.47)	116.90 (26.69)
Yes	32.75 (8.73)	33.04 (11.91)	42.99 (13.44)	108.79 (29.27)
***p-value***	**0.027**	**0.010**	0.131	**0.014**
**Any previous mental health treatment**				
No	34.78 (8.68)	37.08 (11.27)	44.98 (11.31)	116.85 (26.30)
Yes	33.76 (8.68)	34.06 (12.08)	43.94 (12.92)	111.76 (28.88)
***p-value***	0.312	**0.029**	0.466	0.119

**In bold, statistically significant values. *p* = *p*-value. (SD) indicate standard deviation. For *n* (%) of each variable, check Table 1.**

**Table 4:** Results of the multiple linear regression analysis on the mean total and subscale scores of the Support and Control in Birth (SCIB) questionnaire.

Variable	Internal Control aMD (95% CI); *p* N = 302	External Control aMD (95% CI); *p* N = 302	Support aMD (95% CI); *p* N = 301	SCIB Global aMD (95% CI); *p* N = 302
**Age**	—	−0.29 (−0.49, −0.08); **0.006**	—	—
**BSSR (per point)**	0.56 (0.48, 0.64); **<0.001**	0.75 (0.61, 0.88); **<0.001**	0.89 (0.77, 1.01); **<0.001**	2.35 (2.13, 2.56); **<0.001**
**Level of pain during labour (per point)**	−1.00 (−1.31, −0.70); **<0.001**	0.51 (0.14, 0.88); **0.008**	—	—
**Visits to A&E (per additional visit)**	—	—	0.54 (0.05, 1.03); **0.031**	—
**Previous mental health care**	—	−2.40 (−4.14, −0.67); **0.007**	—	—
**Current illness vs. no**	—	—	—	6.38 (1.74, 11.02); **0.007**
**Desired pregnancy vs. not**	—	—	—	—
**Health problems during pregnancy vs. none**	—	1.84 (0.08, 3.60); **0.041**	—	—
**High-risk pregnancy vs. low-risk pregnancy**	—	—	−0.75 (−1.39, −0.10); **0.023**	−5.62 (−10.00, −1.25); **0.012**
**Multiparity vs. primiparity**	−1.43 (−3.07, 0.20); 0.086	—	—	—
**Belongs to a religion Yes and does not practise**	1.13 (−0.51, 2.77); 0.175			
**Belongs to a religion (Yes and practises)**	2.31 (0.20, 4.42); **0.032**			
**Birth plan followed**	—	—	1.71 (−0.25, 3.66); 0.087	3.09 (−0.71, 6.88); 0.110
**Birth plan not followed**	—	—	−7.71 (−10.72, −4.71); **<0.001**	−10.21 (−16.01, −4.42); **0.001**
**Onset of labour: Induced**	—	—	−0.42 (−2.34, 1.49); 0.664	—
**Onset of labour: Scheduled caesarean**	—	—	−5.92 (−10.72, −1.13); **0.016**	—
**Onset of labour: emergency C-section**	—	—	2.76 (−2.59, 8.11); 0.311	—
**Active participation during labour: Not possible**	—	0.86 (−4.56, 6.29); 0.754	—	—
**Active participation**	—	7.15 (2.21, 12.09); **0.005**	—	—
**No epidural use for medical reasons**	0.03 (−6.71, 6.76); 0.994	−0.20 (−7.86, 7.45); 0.958	—	—
**No epidural use for non-medical reasons**	−3.54 (−7.29, 0.20); 0.064	−4.83 (−6.07, −0.42); **0.032**		
**Use of epidural**	−2.57 (−5.03, −0.12); **0.040**	−3.67 (−6.47, −0.87); **0.010**	—	—
**Instrumental delivery**	—	−2.79 (−5.26, −0.32); **0.027**	—	—
**Elective caesarean**		−1.75 (−6.07, 2.57); 0.425		
**Emergency caesarean**	—	−1.31 (−3.98, 1.35); 0.333	—	—
**Model Fit Adjusted R^2^**	0.461	0.622	0.594	0.701
**F test**	33.13; *p* < 0.001	40.88; *p* < 0.001	55.83; *p* < 0.001	141.87; *p* < 0.001
**Durbin-Watson**	1.885	1.884	2.020	1.962

aMD = adjusted mean difference; CI = confidence interval; VIF = variance inflation factor. *p* = *p*-value. Models were fitted using complete-case analysis. Model fit was assessed using R^2^, adjusted R^2^ and the overall F test. Regression diagnostics included Durbin-Watson statistics, tolerance and VIF values, standardised and studentised residuals, Mahalanobis distance, centred leverage values and Cook’s distance. Dashes (—) indicate no significant effect or a variable not included in the final model.

## Data Availability

The data presented in this study are available on reasonable request from the corresponding author.

## Supplementary Materials

The following supporting information can be downloaded at: https://www.mdpi.com/article/10.3390/medicina62071281/s1. Supplementary Table S1. Sensitivity analysis restricted to women with vaginal birth; Supplementary Table S2. Sensitivity analysis excluding BSS-R from the regression models.
