# Supplementary material for: Factors Associated with Perceptions of Maternal Control and Support During Childbirth and Relationship with Childbirth Satisfaction Among Women in Spain
Source: Medicina (Kaunas). 2026 Jul 3;62(7):1281. doi: 10.3390/medicina62071281 (PMC13413871; doi:10.3390/medicina62071281)
Supplement: Supplementary file 1 [file medicina-62-01281-s001.zip › medicina-4349946-supplementary.pdf]

Supplementary Table S1. Sensitivity analysis restricted to women with vaginal birth

Results of multiple linear regression models on total and subscale SCIB scores among women with vaginal birth.

Variable	Internal control aMD (95% CI); p	External control aMD (95% CI); p	Support aMD (95% CI); p	SCIB global aMD (95% CI); p
**BSS-R (per point)**	0.69 (0.55, 0.83); <0.001	0.81 (0.63, 0.99); <0.001	0.82 (0.64, 1.00); <0.001	2.36 (2.01, 2.71); <0.001
**Maternal age (per year)**	—	-0.32 (-0.59, -0.06); 0.017	—	—
**Pain intensity during labour (per point)**	-0.99 (-1.45, -0.53); <0.001	0.37 (-0.19, 0.92); 0.199	—	—
**A&E visits during pregnancy/childbirth (per additional visit)**	—	—	0.29 (-0.39, 0.97); 0.405	—
**Previous mental health care vs. no**	—	-1.91 (-4.22, 0.39); 0.103	—	—
**Current illness vs. no**	—	—	—	5.07 (-1.05, 11.19); 0.104
**Health problems during pregnancy vs. none**	—	2.16 (-0.18, 4.49); 0.070	—	—
**High-risk pregnancy vs. low-risk pregnancy**	—	—	—	-6.95 (-12.91, -0.98); 0.023
**Multiparity vs. primiparity**	-1.62 (-3.55, 0.31); 0.100	—	—	—
**Belongs to a religion: yes, non-practising**	0.99 (-1.09, 3.08); 0.348	—	—	—
**Belongs to a religion: yes, practising**	3.24 (0.65, 5.82); 0.014	—	—	—
**Birth plan followed**	—	—	2.02 (-0.37, 4.41); 0.096	4.11 (-0.53, 8.75); 0.082
**Birth plan not followed**	—	—	-7.43 (-12.95, -1.92); 0.008	-9.21 (-20.04, 1.62); 0.095
**Onset of labour: induced vs. spontaneous onset**	—	—	-1.65 (-4.00, 0.69); 0.165	—
**No epidural use for medical reasons**	0.20 (-6.40, 6.80); 0.953	-0.67 (-8.54, 7.20); 0.866	—	—
**No epidural use for non-medical reasons**	-3.58 (-7.89, 0.73); 0.103	-3.29 (-8.76, 2.19); 0.237	—	—
**Use of epidural**	-2.00 (-4.77, 0.77); 0.155	-4.49 (-7.81, -1.17); 0.008	—	—
**Active participation during childbirth: not possible**	—	-2.28 (-9.38, 4.83); 0.528	—	—
**Active participation during childbirth: yes**	—	4.70 (-1.08, 10.47); 0.110	—	—
**Pregnancy follow-up/control variable**	—	—	-0.58 (-1.38, 0.23); 0.157	—
**Model n**	181	181	180	181
**Adjusted R^2**	0.436	0.438	0.402	0.561
**F test**	18.42; p < 0.001	15.00; p < 0.001	21.06; p < 0.001	47.02; p < 0.001
**Durbin-Watson**	2.007	1.918	1.895	1.932

aMD = adjusted mean difference; CI = confidence interval; BSS-R = Birth Satisfaction Scale-Revised; SCIB = Support and Control in Birth. Dashes indicate that the variable was not included in the corresponding final model or did not apply to the sensitivity model. Models were fitted using complete-case analysis. The sensitivity analysis was restricted to women with vaginal birth; therefore, mode of delivery was constant and was not included in the models.

Supplementary Table S2. Sensitivity analysis excluding BSS-R from the regression models

Results of multiple linear regression models on total and subscale SCIB scores after excluding childbirth satisfaction from the models.

Variable	Internal control aMD (95% CI); p	External control aMD (95% CI); p	Support aMD (95% CI); p	SCIB global aMD (95% CI); p
**Maternal age (per year)**	—	-0.18 (-0.42, 0.06); 0.148	—	—
**Pain intensity during labour (per point)**	-1.31 (-1.69, -0.93); <0.001	-0.11 (-0.54, 0.32); 0.621	—	—
**A&E visits during pregnancy/childbirth (per additional visit)**	—	—	-0.21 (-0.85, 0.44); 0.526	—
**Previous mental health care vs. no**	—	-3.54 (-5.59, -1.48); <0.001	—	—
**Current illness vs. no**	—	—	—	7.55 (0.10, 15.00); 0.047
**Health problems during pregnancy vs. none**	—	1.03 (-1.07, 3.12); 0.335	—	—
**High-risk pregnancy vs. low-risk pregnancy**	—	—	—	-14.67 (-21.57, -7.76); <0.001
**Multiparity vs. primiparity**	-0.43 (-2.48, 1.61); 0.677	—	—	—
**Belongs to a religion: yes**	1.59 (-0.47, 3.65); 0.130	—	—	—
**Belongs to a religion: yes, practising**	1.09 (-1.56, 3.74); 0.417	—	—	—
**Birth plan followed**	—	—	3.22 (0.62, 5.83); 0.015	7.28 (1.22, 13.35); 0.019
**Birth plan not followed**	—	—	-14.52 (-18.36, -10.69); <0.001	-31.80 (-40.57, -23.03); <0.001
**Onset of labour: induced**	—	—	-3.44 (-5.95, -0.93); 0.007	—
**Onset of labour: scheduled caesarean section**	—	—	-12.25 (-18.57, -5.94); <0.001	—
**Onset of labour: emergency C-section**	—	—	-5.01 (-12.03, 2.02); 0.162	—
**No epidural use for medical reasons**	1.41 (-7.07, 9.89); 0.744	-0.38 (-9.51, 8.75); 0.935	—	—
**No epidural use for non-medical reasons**	-5.39 (-10.10, -0.69); 0.025	-3.68 (-8.93, 1.56); 0.168	—	—
**Use of epidural**	-5.21 (-8.26, -2.15); <0.001	-5.29 (-8.61, -1.97); 0.002	—	—
**Mode of delivery: instrumental**	—	-7.76 (-10.51, -5.01); <0.001	—	—
**Mode of delivery: elective caesarean section**	—	-7.35 (-12.37, -2.33); 0.004	—	—
**Mode of delivery: emergency caesarean section**	—	-7.24 (-10.16, -4.32); <0.001	—	—
**Active participation during childbirth; no, not possible**	—	-5.56 (-11.89, 0.77); 0.085	—	—
**Active participation during childbirth: yes**	—	7.53 (1.63, 13.43); 0.013	—	—
**Pregnancy follow-up/control variable**	—	—	-0.67 (-1.53, 0.19); 0.126	—
**Model n**	302	302	301	302
**Adjusted R²**	0.145	0.462	0.274	0.227
**F test**	8.29; p < 0.001	22.55; p < 0.001	17.20; p < 0.001	23.13; p < 0.001
**Durbin-Watson**	1.891	1.934	1.965	1.955

aMD = adjusted mean difference; CI = confidence interval; SCIB = Support and Control in Birth. Dashes indicate that the variable was not included in the corresponding final model. Models were fitted using complete-case analysis. The sensitivity analysis repeated the multivariable models after excluding the BSS-R score because childbirth satisfaction and perceived support/control are conceptually related dimensions of childbirth experience.
